# Supplementary figures and images for: Negative Selection on a SOD1 Mutation Limits Canine Degenerative Myelopathy While Avoiding Inbreeding
Source: Genome Biol Evol. 2023 Dec 18;16(1):evad231. doi: 10.1093/gbe/evad231 (PMC10773665; doi:10.1093/gbe/evad231)

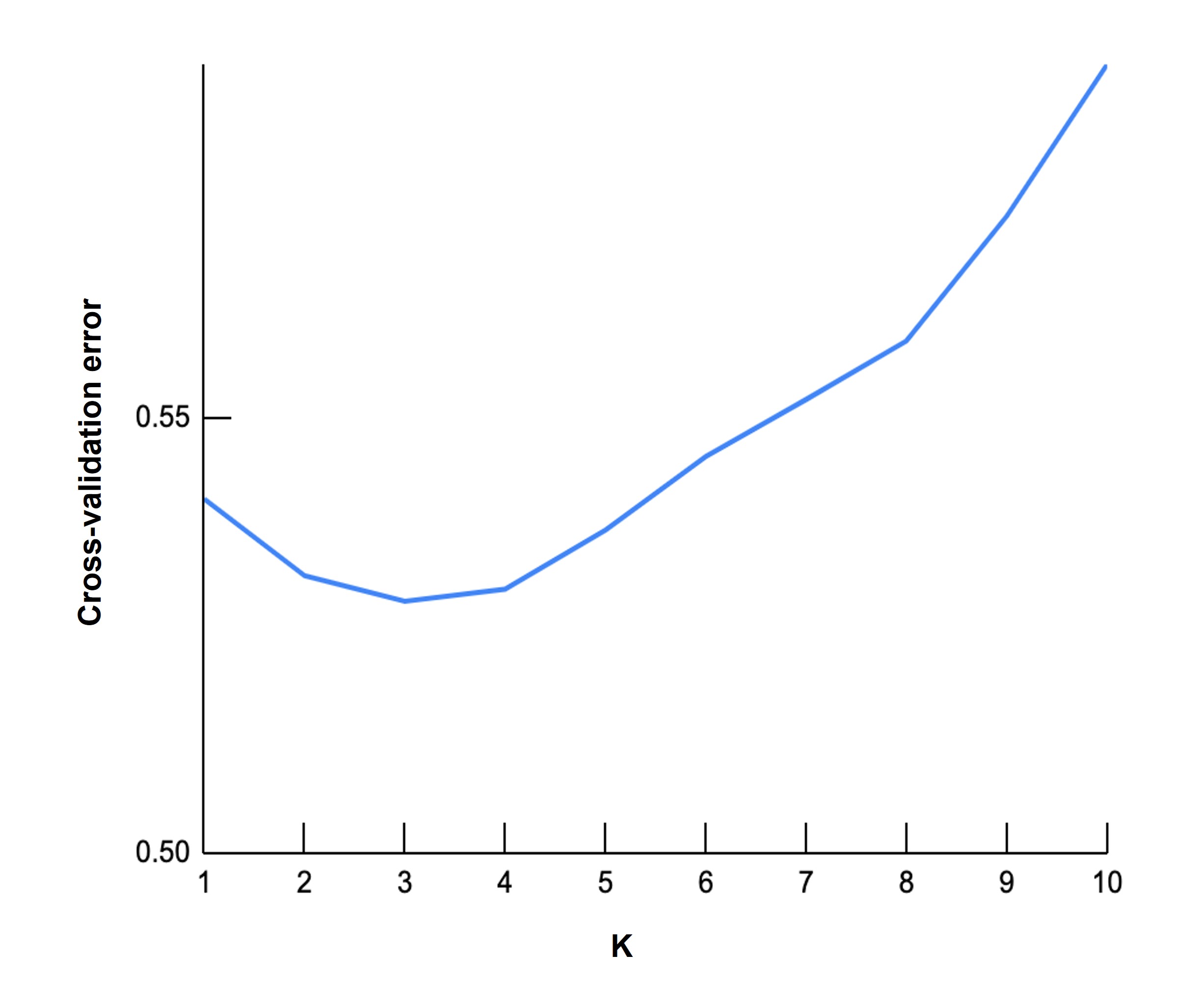

Supplement: evad231_Supplementary_Data [file evad231_supplementary_data.zip › FigS1.jpg]
